# Dynamin-1 is a potential mediator in cancer-related cognitive impairment

**DOI:** 10.1016/j.neurot.2024.e00480

**Published:** 2024-11-07

**Authors:** Ding Quan Ng, Casey Hudson, Tracy Nguyen, Sukesh Kumar Gupta, Yong Qin Koh, Munjal M. Acharya, Alexandre Chan

**Affiliations:** a Department of Clinical Pharmacy Practice, School of Pharmacy & Pharmaceutical Sciences, University of California Irvine, Irvine, CA, USA; b Department of Anatomy & Neurobiology, School of Medicine, University of California Irvine, Irvine, CA, USA; c Department of Radiation Oncology, School of Medicine, University of California Irvine, Irvine, CA, USA; d Department of Oncology Pharmacy, National Cancer Centre Singapore, Singapore

**Keywords:** Cancer-related cognitive impairment, Chemobrain, Dynamin-1, Extracellular vesicle, Exosome

## Abstract

Dynamin-1 (DNM1) is crucial for synaptic activity, neurotransmission, and associative memory, positioning it as a potential biomarker of cancer-related cognitive impairment (CRCI), a neurological consequence of cancer treatment characterized by memory loss, poor concentration, and impaired executive function. Through a stepwise approach, this study investigated the role of DNM1 in CRCI pathogenesis, incorporating both human data and animal models. The human study recruited newly diagnosed, chemotherapy-naïve adolescent and young adult cancer and non-cancer controls to complete a cognitive instrument (FACT-Cog) and blood draws for up to three time points. Following that, a syngeneic young-adult WT (C57BL/6) female mouse model of breast cancer chemobrain was developed to study DNM1 expression in the hippocampus. Samples from eighty-six participants with 30 adolescent and young adult (AYA) cancer and 56 non-cancer participants were analyzed. DNM1 levels were 32 ​% lower (P ​= ​0.041) among cancer participants compared to non-cancer prior to treatment. After receiving cytotoxic treatment, cognitively impaired cancer patients were found to have 46 ​% lower DNM1 levels than those without impairment (P ​= ​0.049). In murine breast cancer-bearing mice receiving chemotherapy, we found a greater than 40 ​% decline (P ​< ​0.0001) in DNM1 immunoreactivity in the hippocampal CA1 and CA3 subregions concurrent with a deterioration in spatial recognition memory (P ​< ​0.02), compared to control mice without exposure to cancer and chemotherapy. Consistently observed in both human and animal studies, the downregulation of DNM1 is linked with the onset of CRCI. DNM1 might be a biomarker and therapeutic target for CRCI.

## Introduction

Cancer-related cognitive impairment (CRCI) is a complication of cancer and associated treatments and encompasses cognitive symptoms, including difficulties with memory, poor concentration, and executive functioning [1,2]. Affected patients report challenges with resuming pre-cancer responsibilities at work, school, and home, which markedly reduce their quality of life [[3], [4], [5]]. To date, there remains a lack of effective pharmacological interventions that can be routinely provided to these patients.

Dynamin-1 (DNM1), a protein maximally expressed in the central nervous system [6], plays a pivotal role in memory formation by facilitating effective neurotransmission and synaptic plasticity [[7], [8], [9], [10]], and lower DNM1 concentrations have been observed in post-mortem brains of dementia patients [11]. As long-term memory impairment is also an important sign of CRCI, downregulation in DNM1 may be associated with CRCI pathogenesis.

We previously reported a greater downregulation in DNM1 levels in plasma extracellular vesicles (EVs) proteomes of breast cancer survivors with perceived cognitive impairment [12]. EVs mediate intercellular communication and may contribute to disease pathogenesis, suggesting DNM1 as a potential mediator in CRCI [13]. The study, however, was limited by the use of pooled participant samples and was thus unable to account for characteristics that could confound the DNM1-CRCI relationship when measuring subjective cognition (e.g. co-occurring psychiatric comorbidities [14]). Another important question is the representativeness of DNM1 levels in plasma EVs with brain activity. It is thus necessary to understand how DNM1 expression changes in the brain with tumor induction and chemotherapy administration to develop a more comprehensive picture of the DNM1-CRCI theory.

This study utilized stepwise clinical and pre-clinical approaches to enhance our understanding of the DNM1-CRCI relationship that we observed in our exploratory study [12]. First, we investigated the presence of differential DNM1 expression between cancer patients with and without perceived CRCI (adjusted for confounders) in an existing prospective cohort of newly diagnosed adolescent and young adult (AYA) cancer patients [15]. The choice to study the DNM1-CRCI relationship in AYA patients helped reduce potential confounding effects due to aging, which are often implicated in CRCI studies. This was followed by an animal study that compared cognitive function and DNM1 expression in the hippocampus between non-cancer and cancer-bearing, chemotherapy-exposed mice to evaluate brain-associated changes in DNM1 expression due to cancer and chemotherapy that could not be feasibly studied in human subjects. We hypothesize that DNM1 expression is significantly downregulated among AYA cancer patients suffering from CRCI and within the hippocampus of cancer-bearing, chemotherapy-treated mice.

## Materials and Methods

### Human study

#### Study design

This study was nested within the Adolescent and Young Adult Cancer Patients: Cognitive Toxicity on Survivorship (ACTS) study, a prospective, longitudinal cohort study, conducted in Singapore from 2018 to 2022 (NCT03476070). This cohort was originally created to investigate the incidence of cognitive impairment in AYA cancer patients before, during and after anti-cancer treatment. We received ethics approval from SingHealth Institutional Review Board (CIRB Ref 2017/3139) and all participants provided informed consent before participation. Detailed characteristics are previously described in Chan et al. [15]

#### Patient eligibility criteria

The ACTS study recruited two participant groups. The first group comprised **AYA cancer patients** who were 15–39 years old, newly diagnosed with cancer, and treatment naïve. The second group were **non-cancer community controls** who were age-matched (3-year intervals) to cancer participants, did not have prior cancer diagnosis, and were not immediate family members to any of the cancer participants. Participants were excluded if there was evidence of psychosis or neuropsychiatric illness that impaired cognitive functioning. In this study, we focused on participants who completed all required questionnaires and blood draws in at least two assessment time points in order to facilitate complete case analysis.

#### Assessment time points and procedures

Cancer patients were evaluated at three time points (pre-treatment, 3-, and 6-months post-baseline, representing T1, T2 and T3 respectively). Non-cancer controls completed two time points (T1 and T3). All participants completed questionnaires in-person, facilitated by a trained research assistant, for evaluating perceived cognitive function (FACT-Cog version 3) and neuropsychiatric symptoms (MFSI-SF and RSCL psychological distress [RSCL-PD]) and provided blood samples at all time points. Higher FACT-Cog total scores represent better perceived cognitive function, while higher MFSI-SF total and RSCL-PD subscale scores represent worse fatigue and distress, respectively. Sociodemographic and cancer-related characteristics were collected from baseline surveys and medical records [15,16].

#### Perceived cognitive decline

Participants with perceived cognitive decline (PCD) were identified based on clinically important difference of ≥10.6-point decline in FACT-Cog total score (range 0–148) from baseline [17].

#### Plasma collection

9 ​ml of blood was drawn, stored in ethylenediaminetetraacetic acid tubes, and then centrifuged at 1069 ​× ​*g* for 10 ​min at 4 ​°C by trained personnel. Plasma was aliquoted into cryotubes and stored in a −80 ​°C freezer until analysis.

#### Extracellular vesicle isolation

Plasma EVs are isolated using differential ultracentrifugation as previously described [12,18]. In brief, the plasma samples collected were diluted with an equal volume of phosphate-buffered saline (PBS) and centrifuged at 2000 × *g* for 30 min and 12,000 *× g* for 60 min, both at 4 °C to remove cells and cellular debris. The isolated plasma EVs were obtained by ultracentrifugation at 100,000 *× g* for 18 h using a Beckman L100-XP Ultracentrifuge and stored at −80 °C until further analysis.

#### Extracellular vesicle visualization

The enriched plasma EVs were prepared for transmission electron microscopy (TEM) visualization by transferring them onto a copper grid (Electron Microscopy Services, USA), washed in water and fixed with 2.5 ​% glutaraldehyde (Electron Microscopy Services, USA) for 10 ​min. Subsequently, negative staining was performed by incubating with 2.5 ​% gadolinium triacetate (Electron Microscopy Services, USA) for 2 ​min. The samples were then viewed using the FEI Tecnai 12 TEM, operating at 100 ​kV (FEI, USA) [12].

#### Evaluation of Dynamin-1 protein concentration in plasma EVs

The protein concentration of DNM1 and Flotillin-1 were quantified using commercially available enzyme-linked immunosorbent assay (ELISA) kits (ABclonal RK01275 and FineTest EH1351 respectively, USA) [19]. The DNM1 protein concentration in the plasma EVs were evaluated by normalizing DNM1 against Flotillin-1 levels. DNM1 levels are presented as pg/ng, which reports the amount of DNM1 in picograms per nanogram of Flotillin-1.

#### Descriptive statistics

For categorical variables, group characteristics were compared using the Chi-square test (expected cell counts ≥5), or Fisher's exact test (expected cell counts <5). For continuous variables, the independent *t*-test was used if the data were normally distributed and had equal variances (presented as mean and standard deviations), while the Mann-Whitney *U* test was applied for non-normally distributed data or when the assumption of equal variances was violated (presented as median and interquartile ranges). The distribution of DNM1 levels was examined with histogram plots. Ethnicity is summarized using the “Chinese, Malay, Indian and Others” categorization commonly used in Singapore [15].

#### Univariate analysis – relationship between Dynamin-1, cancer, neurotoxic therapies and cognitive decline

The Spearman correlation test was conducted to evaluate the relationship between the changes in DNM1 expression and FACT-Cog scores from baseline. The effect of cancer and exposure to neurotoxic chemotherapy [[20], [21], [22]] (pre-, on-, and post-treatment) on DNM1 levels were evaluated with generalized estimating equation (GEE) using a sandwich variance estimator, gaussian family, log link function (for log-transformation of non-normally distributed DNM1 data) and an exchangeable correlation matrix to account for repeated measures and working with non-normally distributed biological measures (non-normal, positively skewed distribution). Additionally, the differences in post-baseline DNM1 levels between PCD and non-PCD (reference group) patients were evaluated using GEE for cancer patients (after treatment exposure, collected at two timepoints) and generalized linear model (GLM) for non-cancer controls (collected at one timepoint), both with log-transformed DNM1 levels, and adjusted for baseline DNM1.

#### Multivariate analysis

Population-averaged differences in post-baseline DNM1 levels were compared between PCD and non-PCD participants within each patient strata (cancer or non-cancer) with GEE as described previously. Confounders include baseline DNM1 levels, cancer status (yes/no), age, gender, marital status, education years, and changes in psychological distress (RSCL-PD subscale) and fatigue (MFSI-SF total score) from baseline. PCD status was designed to be a time-varying variable (e.g. participants with PCD at T2 may not have PCD in T3) due to known heterogeneous trajectories of cognitive function among cancer survivors [23]. All observations were included to facilitate the precise estimation of coefficients. Differences in post-baseline DNM1 levels were calculated based on linear combinations of the following coefficients for each patient strata.

• Non-cancer participants: PCD status.
• Cancer patients: PCD status, PCD ​× ​cancer. The explanation for these calculations is documented in the Supplement (Table S1).

All coefficients were exponentiated to account for the use of log link function, presented as relative changes (RCs), and accompanied by 95 ​% confidence intervals and p-values. To improve interpretability of the model output, marginal differences (Δ) in DNM1 levels attributable to PCD were predicted within each cancer and non-cancer strata using the fitted GEE model.

#### Exploratory analysis

As our animal study was performed on a breast cancer model exposed to adriamycin (doxorubicin) and cyclophosphamide (ADR-CYP), we conducted an exploratory analysis among cancer patients to evaluate the impact of breast cancer, ADR-CYP regimen, and their interaction on DNM1 levels with the hypothesis that these exposures do not differentially affect DNM1 levels. The same model was also used to evaluate the durability of the differences in DNM1 levels between those with and without PCD among cancer patients.

All analyses were tested at a 5 ​% significance level and completed on Stata version 16.1 (College Station, Texas, USA).

### Animal study

#### Mouse model

All experimental procedures followed National Institutes of Health (NIH) vertebrate animal use guidelines and were approved by the University of California Irvine (UCI) Institutional Animal Care and Use Committee (AUP-22-144). 4 months old C57BL/6 wild-type (WT) female mice (Jackson Laboratory, USA) were housed and maintained on a 12:12 ​h light:dark cycle with free access to food and water ad libitum.

#### Breast cancer induction

Py230-luc (luciferase expressing) murine breast cancer cells were cultured as a monolayer at 37 ​°C with 5 ​% CO2 in an incubator (Thermo Fisher Scientific, USA) in F–12K medium (Thermo Fisher Scientific, USA) supplemented with 10 ​% fetal bovine serum (FBS, Gibco), and 0.1 ​% MITO+ ​serum extender (Corning) and 15 ​% Penn-Strep (Gibco). On the day of tumor implantation, Py230-luc cells were harvested as recommended (ATCC) and prepared as 5 ​× ​10^5^ ​cells in a 50 ​μl hibernation buffer (Hibernate-A medium, Gibco) and 20 ​% polyethylene glycol (PEG, Thermo Fisher Scientific). Tumor cells were implanted in the third mammary gland of each mouse (N ​= ​8 mice/group). Hairs were removed using a body cream hair remover (Nair). Py230 ​cells or buffer were injected directly into the mammary fat pad through a tweezer-uplifted nipple using a 26-gauge needle. The day following injections the mice received 100 ​% aloe vera (Fruit of the Earth) on the injection site to soothe the skin. Animals were monitored closely for one-week post-injection. We did not observe any adverse reaction, and the injection site was completely healed within 2–3 days. Control mice received sham injection treatment using hibernation buffer as above.

#### Breast cancer monitoring

We used two methods to measure tumor growth: digital caliper and bioluminescence imaging (BLI). First, the tumor growth was measured using a digital caliper, and tumor volumes were calculated as ellipsoid volume (1/6 π ​× ​L ​× ​W ​× ​(L ​+ ​W)/2) weekly over eight weeks as described [24]. Concurrently, *in vivo* tumor detection and volume measurements were facilitated by BLI following luciferin injection (GoldBio). Briefly, animals were injected with 0.2 ​ml (IP) D-luciferin sodium salt (15 ​mg/ml in PBS). Mice were anesthetized using 2.5 ​% v/v inhalant isoflurane gas anesthesia (VetEquip RC2). Mice resting in a prone position inside the chamber of the imager (IVIS Lumina III, PerkinElmer) were imaged at a peak emittance time of 12 ​min post-injection. Tumor volumes were reported as relative bioluminescence (photons/s).

#### Chemotherapy preparation and administration

1–2 days after the confirmation of tumor growth a cohort of mice was administered a chemotherapy schedule (N ​= ​8 mice/group). Mice were treated with an adjuvant chemotherapy regimen using adriamycin (ADR, doxorubicin hydrochloride, Sigma) and cyclophosphamide (CYP, Cytoxan, Selleckchem). Mice received ADR made in deionized water at a dose of 2 ​mg/kg (once weekly, intra-peritoneal, IP) over the course of four weeks. One hour after ADR injections, mice received an injection of CYP made in saline at a dose of 50 ​mg/kg (once weekly, IP) for four weeks. ADR and CYP were made as injectable solutions at the beginning of the experiment, and they were then aliquoted and immediately frozen at −20 ​°C until use. The therapeutic response of tumors to chemotherapy was monitored weekly using the digital caliper and BLI as above. In parallel, noncancer WT female mice received ADR-CYP for comparison of DNM1 expression with the cancer-bearing mice.

#### Cognitive function assessment – object location memory (OLM) task

Mice were tested on an OLM task 7–8 weeks after their final chemotherapy treatment (N ​= ​8 mice/group). OLM evaluates spatial recognition memory, which is dependent on unimpaired hippocampal function [25,26]. The test was conducted over four days: three days for arena habituation and one test day. The arena was a white box (33 ​× ​33 ​× ​33 ​cm) with a fresh layer of corn cob bedding that was replaced after every run. Blue tape was placed on one wall of the testing arena to create a recognizable reference to the spatial location. Habituation and test day occurred in a dimly lit room (50–60 lux). The animal's behavior was recorded using a ceiling-mounted camera, and live tracking was recorded using a Noldus Ethovision XT system (v17.0, Noldus Information Technology, Inc., Leesburg, VA, USA). Arena habituation was conducted for 10 ​min per day in an empty arena with bedding to habituate the mice to the testing environment. On test day, two similar lego block toys (objects) were placed on the same side of the arena closest to the blue tape (familiarization phase). For the testing phase, one of the toys was moved to the opposite side of the arena. During test day, mice were placed into the arena for the familiarization phase for 5 ​min; they were then returned into their home cage in a dark room for 5 ​min and then re-introduced into the arena for a 5-min testing phase. During habituation and test day the mice were video recorded, and time spent exploring was captured. The videos were scored for time spent interacting with the toys (nose point directed to the object). For the statistical analysis of this data, a memory index (MI) was the main dependent variable. MI was calculated as the proportion of total time spent exploring the novel spatial location [MI = ((t_novel_/t_total_) – (t_familiar_/t_otal_)) x 100, where t is time]. Data is presented as mean ​± ​SEM (N ​= ​8 mice per group), *P-*values were derived from unpaired two-tailed *t*-test and the analysis was considered significant for a value of *P* ​≤ ​0.05.

#### Mouse perfusion and brain tissue collection

After completion of the behavior study (8–9 weeks post-tumor induction), animals were euthanized by intracardiac perfusion using PBS with heparin (10U/mL, 100 ​mM, pH 7.4, Sigma) and 4 ​% paraformaldehyde made in PBS (100 ​mM, pH 7.4, Sigma). Fixed brains were immersed in a gradient of sucrose solutions (10–30 ​%, Sigma) and sectioned coronally (30 ​μm thick) using a cryostat (Microm, Epredia). Sections were collected and stored in PBS (Gibco) with sodium azide (0.02 ​%) at 4 ​°C. Free-floating hippocampus-containing coronal brain sections were selected at −2.00 to −2.90 ​mm posterior to bregma. DAPI nuclear counterstaining facilitated the identification of hippocampal layers, including the dentate gyrus, CA1, and CA3 subregions.

#### Dynamin-1 immunofluorescence staining and volumetric analysis

Brain sections were washed with PBS (Gibco) containing 0.3 ​% Tween-20 (Sigma) and then incubated in the 3 ​% hydrogen peroxide (Sigma) solution in PBS and washed with PBS. Sections were then blocked with 10 ​% normal donkey serum (NDS, Jackson Research) in PBS and 0.01 ​% Triton (Sigma) for 45 ​min and then with a primary antibody solution in PBS (rabbit anti-DNM1, 1:250; Synaptic Systems) overnight at 4 ​°C on a temperature-controlled see-saw shaker (Enviro-Genie Scientific). Subsequently, sections were washed with PBS and incubated with donkey anti-rabbit Alexa Fluor 568 secondary antibody (Abcam, 1:200) made in PBS with 3 ​% NDS for 1 ​h. Sections were washed with PBS, nuclear counterstained using DAPI (15 ​μM in PBS, Invitrogen), washed again in PBS, and mounted on super frost slides using the Slow Fade Antifade Gold mounting medium (Invitrogen).

The immunofluorescent sections were imaged using a laser-scanning confocal microscope (Nikon AX, Japan) equipped with a 40× ​oil-immersion objective lens (Nikon Plan Apochromat Lambda D, Japan). High-resolution z stacks were acquired using a Nikon Elements AR module at 0.5 ​μm intervals through the brain section. Unbiased deconvolution for the fluorescent z stacks and *in silico* volumetric analyses was carried out using our analytical methods as described [27]. The fluorescence signal was deconvoluted to resolve the optimal fluorescence signal at 568 ​nm wavelength using an adaptive, blinded 3D deconvolution method (ClearView, Imaris v10, Andor Technologies). A 3D algorithm-based surface module (Imaris v10, Andor Technologies) was utilized to evaluate the deconvoluted IMS format images for the volumetric quantification of DNM1 positive immunofluorescent puncta in the hippocampal CA1 and CA3 pyramidal (pyr) and stratum radiatum (sr) subregions. All the Imaris-based data were analyzed using automated batch-processing modules. DNM1 data was expressed as the percentage volume of immunoreactivity to controls. All the immunofluorescence data are expressed as the mean ​± ​SEM. Statistical analyses of these data were conducted using two-way ANOVA (GraphPad Prism, v8.0) and Tukey's multiple comparisons tests. All statistical analyses were considered significant for a value of *P* ​≤ ​0.05.

## Results

### Human study

#### Descriptive statistics

Amongst 75 cancer and 118 non-cancer recruited participants, samples from 30 cancer and 56 non-cancer participants were eligible. Compared to non-cancer, cancer participants received less education and reported a greater degree of psychological distress (P ​< ​0.05). Most were diagnosed with breast (33 ​%) and head/neck cancers (20 ​%) and underwent systemic treatment with platinum agents (63 ​%) and taxanes (37 ​%).

As with other biological measures, DNM1 levels presented a non-normal, positively skewed distribution (Fig. S1). The median DNM1 levels at baseline (pg/ng of Flotillin-1) was significantly lower among cancer participants (29.7 vs 46.1, P ​= ​0.004). This difference remained statistically significant after log-normal GLM adjusted for age, education years, and psychological distress, interpreted as cancer patients having baseline DNM1 levels that were 32 ​% lower than non-cancer controls (RC ​= ​0.68, 95 ​% CI ​= ​0.47 to 0.98, P ​= ​0.041).

At T2, there were 7 (26 ​%) cancer who were with PCD. At T3, there were 8 (27 ​%) cancer and 10 (18 ​%) non-cancer participants reporting PCD. Four (13 ​%) cancer participants experienced PCD at both T2 and T3 (Table 1).

**Table 1:** Descriptive statistics comparing cancer with non-cancer participants.

N		Cancer	Non-cancer	P
		30	56	
**Sex at birth, n (%)**	**Female**	20 (67 ​%)	40 (71 ​%)	0.650
	**Male**	10 (33 ​%)	16 (29 ​%)	
**Age (years)**	**Median (Q1, Q3)**	35.0 (30, 37)	32.5 (27, 35)	0.073
**Ethnicity, n (%)**	**Chinese**	23 (77 ​%)	42 (75 ​%)	0.280
	**Malay**	3 (10 ​%)	2 (4 ​%)	
	**Indian**	1 (3 ​%)	8 (14 ​%)	
	**Others**	3 (10 ​%)	4 (7 ​%)	
**Marriage, n (%)**	**Yes**	16 (53 ​%)	24 (43 ​%)	0.374
	**No**	14 (47 ​%)	32 (57 ​%)	
**Years of education**	**Mean (SD)**	15.6 (3.3)	17.1 (3.0)	0.028∗
**Cancer type, n (%)**	**Breast**	10 (33 ​%)	–	–
	**Head and neck**	6 (20 ​%)	–	
	**Gynecological**	6 (20 ​%)	–	
	**Sarcoma**	3 (10 ​%)	–	
	**Lymphoma**	2 (7 ​%)	–	
	**Colorectal**	1 (3 ​%)	–	
	**Lung**	1 (3 ​%)	–	
	**Testicular**	1 (3 ​%)	–	
**Cancer stage, n (%)**	**I**	6 (21 ​%)	–	–
	**II**	9 (32 ​%)	–	
	**III**	6 (21 ​%)	–	
	**IV**	7 (25 ​%)	–	
**Neurotoxic treatment, n (%)**	**Platinum**	19 (63 ​%)	–	–
	**Rad ​+ ​Chemo**	14 (47 ​%)	–	
	**Taxanes**	11 (37 ​%)	–	
	**Cyclophosphamide**	8 (27 ​%)	–	
	**Adriamycin (doxorubicin)**	7 (23 ​%)	–	
**ECOG, n (%)**	**0**	27 (90 ​%)	–	–
	**1**	3 (10 ​%)	–	
**Baseline Dynamin-1 levels (pg/ng)**	**Median (Q1, Q3)**	29.7 (14.1, 42.2)	46.1 (27.8, 64.4)	0.004∗∗
**Baseline psychological distress (RSCL-PD subscale score**^a^**)**	**Mean (SD)**	13.6 (3.7)	10.7 (3.3)	<0.001∗∗∗
**Baseline fatigue (MFSI-SF total score**^a^**)**	**Mean (SD)**	4.7 (14.5)	−0.6 (17.0)	0.133
**Perceived cognitive decline, n (%)**	**3 months from baseline (T2)**	7 (26 ​%)^b^	–	–
	**6 months from baseline (T3)**	8 (27 ​%)	10 (18 ​%)	0.497

Abbreviations: CRCI, cancer-related cognitive impairment; ECOG, Eastern Cooperative Oncology Group Performance Status; MFSI-SF, Multidimensional Fatigue Symptom Inventory-Short Form; N or n, counts; pg/ng, picogram per nanogram of Flotillin-1; Q1, quartile 1; Q3, quartile 3; Rad ​+ ​Chemo, radiotherapy with chemotherapy; RSCL-PD, Rotterdam Symptom Checklist – Psychological Distress; SD, standard deviation.

∗P ​< ​0.05; ∗∗P ​< ​0.01; ∗∗∗P ​< ​0.001.

a Higher scores represent worse symptoms.

b Of the 30 participants with cancer, 27 (90 ​%) completed all three questionnaires and blood draws at timepoint 2 (T2, 3 months from baseline).

#### Extracellular vesicle characterization

**Fig. 1:**
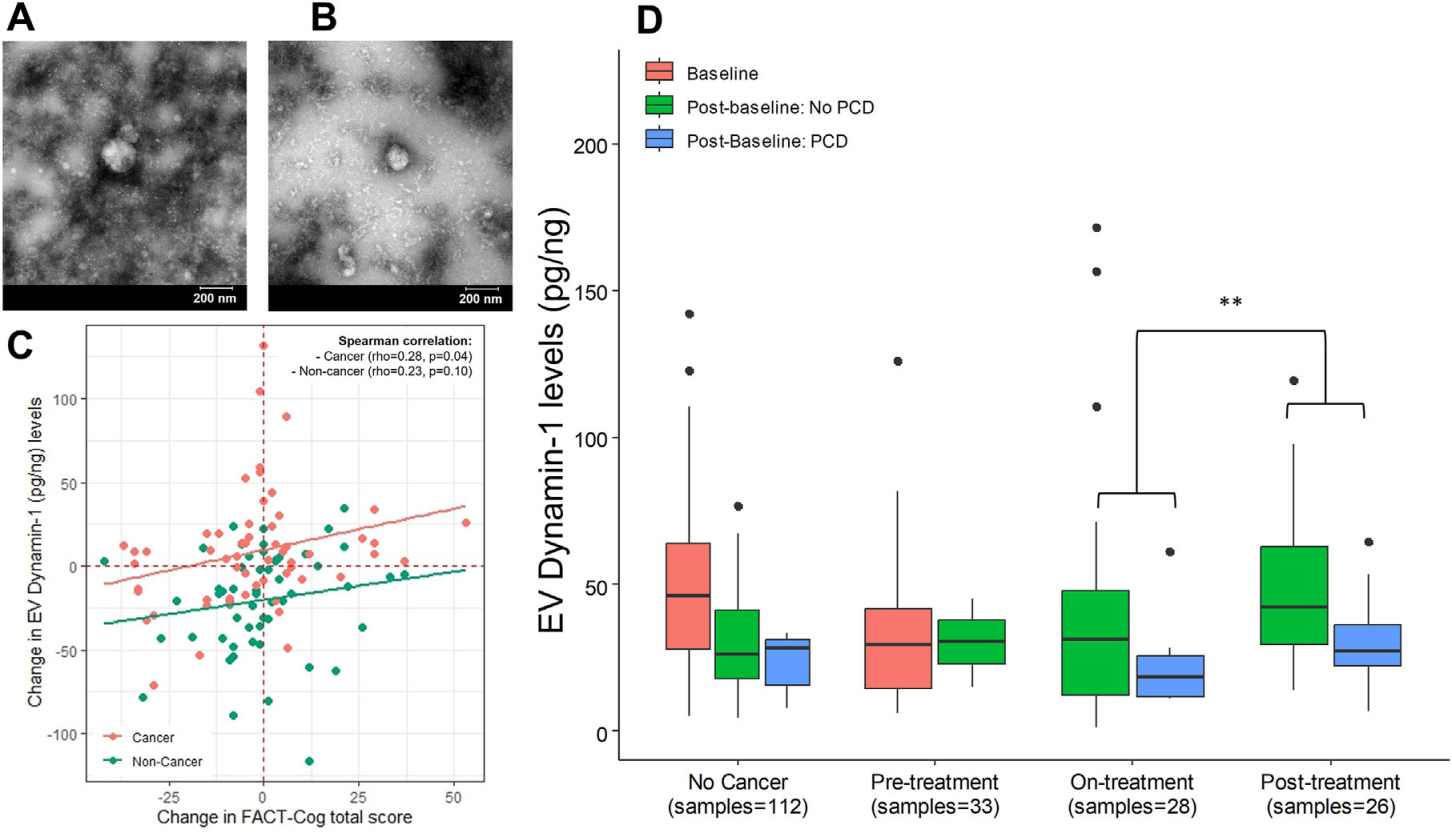
**Extracellular vesicle characterization and differential Dynamin-1 expression from human plasma samples stratified by cancer and perceived cognitive decline statuses.** A representative electron micrograph of isolated human plasma vesicles of **(A)** cancer and **(B)** non-cancer participants showing the spherical morphology of EVs. Scale bars, 200 ​nm **(A**–**B)**. **(C)** Scatter plot correlation between the changes in Dynamin-1 levels (picogram per nanogram of EV marker Flotillin-1) and FACT-Cog total score from baseline. **(D)** Boxplots of Dynamin-1 levels, stratified by cancer and exposure to neurotoxic treatment. Univariate analysis comparing samples from non-cancer, pre- (reference), on-, and post-treatment were performed with generalized estimating equations (GEE), to account for repeated measures, with gaussian distribution and log link function. Analyses did not find significant differences in DNM1 levels between samples collected among non-cancer and cancer patients, regardless of exposure to neurotoxic therapies. Thereafter, differences in post-baseline Dynamin-1 levels between perceived cognitive decline (PCD) and non-PCD (reference) patients were evaluated using GEE for cancer patients (after treatment exposure) and log-normal generalized linear models (GLM) for non-cancer controls, adjusted for baseline Dynamin-1. Dynamin-1 levels were 46 ​% lower among PCD compared to non-PCD cancer patients after exposure to neurotoxic treatment, a difference that is statistically significant (RC ​= ​0.54, 95 ​% CI ​= ​0.37 to 0.81, P ​= ​0.002), but not among controls (RC ​= ​0.77, 95 ​% CI ​= ​0.52 to 1.12, P ​= ​0.174). ∗∗P ​< ​0.01 (**D**).

Plasma EVs displayed spherical morphology (Fig. 1A–B) and the presence of the EV marker Flotillin-1. These findings collectively validate the characterization of these plasma EVs.

#### Univariate analysis – relationship between Dynamin-1, cancer, and cognitive decline

The Spearman correlation coefficient between changes in DNM1 and FACT-Cog total scores was statistically significant among cancer participants (rho ​= ​0.28, P ​= ​0.04), but not among non-cancer controls (rho ​= ​0.23, P ​= ​0.10) (Fig. 1C). GEE analyses did not find significant differences in DNM1 levels between non-cancer and cancer patients, regardless of the exposure to potentially neurotoxic therapies (Fig. 1D). DNM1 levels were 50 ​% lower among PCD compared to non-PCD cancer patients after exposure to neurotoxic treatment, a difference that is statistically significant (RC ​= ​0.50, 95 ​% CI ​= ​0.31 to 0.79, P ​= ​0.003), but not among controls (RC ​= ​0.78, 95 ​% CI ​= ​0.49 to 1.24, P ​= ​0.291) (Fig. 1D).

#### Multivariate analysis

By adjusting for confounders with GEE, cancer patients without PCD, on average, were found with higher DNM1 levels post-baseline compared to non-cancer counterparts (RC ​= ​2.04, 95 ​% CI ​= ​1.51 to 2.77, P ​< ​0.001, Table S1). Further, cancer patients with PCD were found with 46 ​% lower DNM1 levels relative to those without PCD (RC ​= ​0.54, 95 ​% CI ​= ​0.29 to 1.00, P ​= ​0.049, Table 2 – Model A). This difference, between PCD and non-PCD participants, was not observed among non-cancer controls (P ​= ​0.592, Table 2 – Model A). Marginal analyses revealed a statistically significant decline in DNM1 levels attributable to PCD among cancer patients (Δ ​= ​−20.6, 95 ​% CI ​= ​−36.8 to −4.3, P ​= ​0.013) but not among non-cancer controls (Δ ​= ​−3.2, 95 ​% CI ​= ​−14.3 to 8.0, P ​= ​0.576). Ethnicity and fatigue were significantly associated with DNM1 levels (P ​< ​0.05, Table S1).

**Table 2:** Estimated adjusted differences in DNM1 levels, comparing between cognitively impairment and non-impaired participants, using generalized estimating equation with confounder adjustment.

		Differences in post-baseline DNM1 (pg/ng) between cognitively impaired and non-impaired (ref) participants		
		Relative change	95 ​% CI	P
**Model A: All participants**^a^	**Non-cancer**	0.85	0.48 to 1.52	0.592
	**Cancer**	0.54	0.29 to 1.00	0.049∗
**Model B: Cancer patients only (additionally adjusting for breast cancer,** ADR-CYP **exposure, and their interactions)**^b^		0.68	0.43 to 1.08	0.103

Abbreviations: ADR-CYP, adriamycin-cyclophosphamide regimen; CI, confidence interval; pg/ng, picogram per nanogram of Flotillin-1; PCD, perceived cognitive decline; ref, reference.

∗P ​< ​0.05.

a Using both non-cancer and cancer participants' data with post-baseline, log-transformed DNM1 levels as the dependent variable, and adjusted for baseline DNM1 levels, cancer status, age, gender, marital status, education years, as well as change in psychological distress and fatigue levels. The relative differences between non-cancer patients with and without PCD were computed using the exponential of the coefficient for PCD status. The relative differences among cancer patients were computed using the linear combination of PCD status and PCD ​× ​cancer. The explanation of these calculations can be found in the Supplement Table S1.

b Using cancer participants' data with post-baseline, log-transformed DNM1 levels as the dependent variable, and adjusted for baseline DNM1 levels, age, gender, marital status, education years, change in psychological distress and fatigue levels, as well as breast cancer, ADR-CYP exposure and their interactions. The relative differences between cancer patients with and without PCD were computed using the exponential of the coefficient for PCD status.

#### Exploratory analysis

Cancer patients with breast cancer or exposed to the ADR-CYP regimen were not found with different DNM1 levels compared to other cancer patients (P ​> ​0.05, data not reported), suggesting that a rodent model for breast cancer and ADR-CYP exposure is possibly representative of this AYA cohort with regards our investigation into DNM1 activity in the hippocampus. While the difference in DNM1 levels between cancer patients with and without PCD was no longer statistically significant in this model, considering the smaller sample size and statistical power of this analysis, the direction of this difference is consistent with the main findings (Table 2 – Model B).

### Animal study

#### Breast cancer-bearing mice treated with adjuvant chemotherapy were cognitively impaired on a spatial recognition memory task

**Fig. 2:**
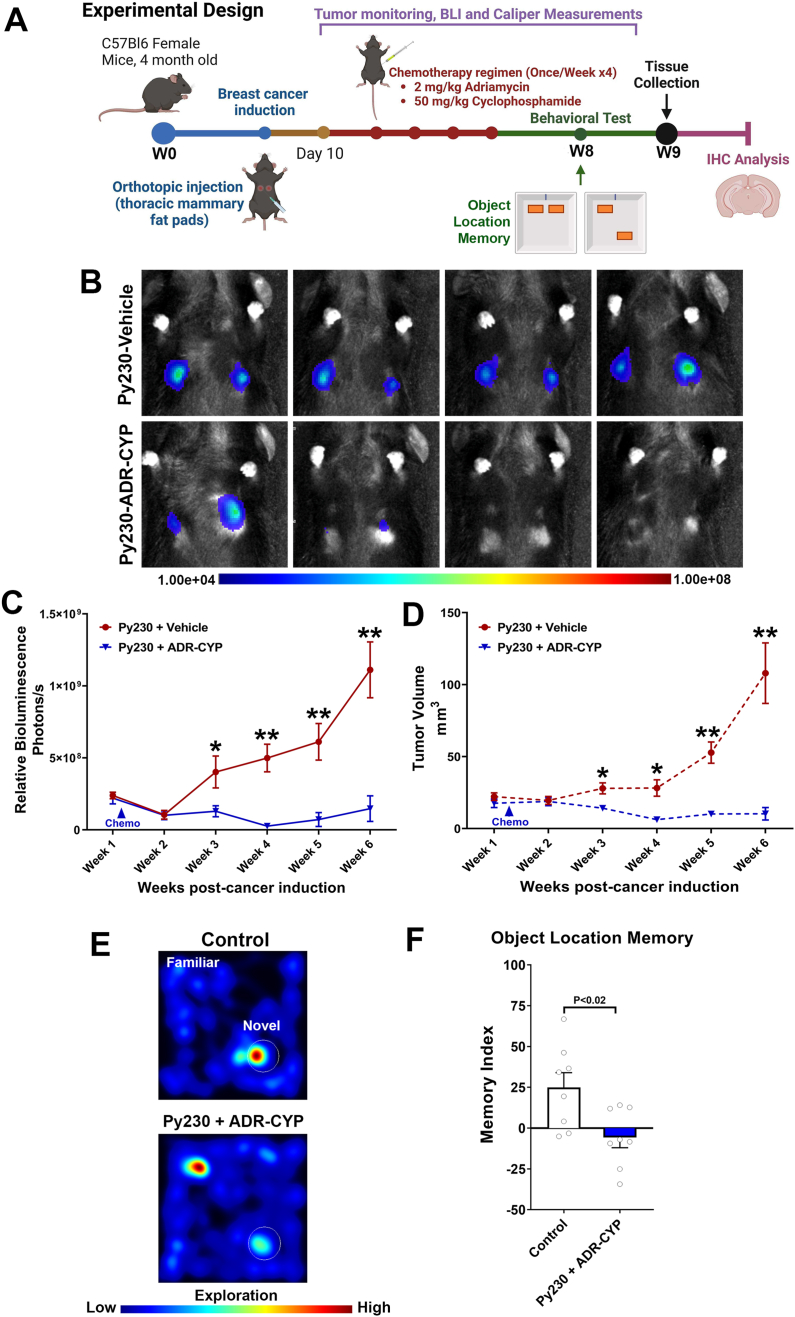
**Adjuvant chemotherapy leads to cognitive decline in breast cancer-bearing mice. (A)** Animal experimental design: Adult WT female mice received an orthotopic injection of murine Py230-luc breast cancer cells or vehicle in the mammary fat pads, and tumor growth was measured using bioluminescence imaging (BLI) and a digital caliper for 6 weeks post-injection. At 10 days post-tumor induction, a cohort of breast cancer-bearing mice received adjuvant chemotherapy, including Adriamycin (ADR, doxorubicin), 2 ​mg/kg, and cyclophosphamide (CYP), 50 ​mg/kg, 1 ​h apart, once weekly for 4 weeks. Controls and Py230 breast cancer-bearing mice receiving adjuvant chemotherapy (ADR-CYP) underwent an object location memory function test (OLM) 8 weeks post-cancer induction. Animals were then euthanized, and brains were collected for the immunofluorescence analysis. **(B**–**D)** Vehicle-treated breast cancer-bearing mice showed a steady increase in the tumor volume as detected by the increased signal *in vivo***(B)** measured as a relative bioluminescence (Photons/s, **C**). Concurrently, caliper measurements of tumor sites showed an increase in the ellipsoid volume (mm^3^, **D**). Adjuvant chemotherapy (ADR-CYP) significantly reduced tumor volume and growth *in vivo***(B**–**D),** thus presenting a breast cancer chemobrain model. **(E)** Representative heat maps showing mice exploring novel (circle) or familiar locations of objects during the OLM task. **(F)** The breast cancer-bearing mice receiving adjuvant chemotherapy (Py230 ​+ ​ADR-CYP) showed a significantly lower preference for the novel placement of the object compared to the control (P ​*<* ​0.02). ∗P ​< ​0.05, and ∗∗P ​< ​0.01 compared to Py230 ​+ ​ADR-CYP **(C**–**D)**. Data is presented as mean ​± ​SEM (N ​= ​8 mice per group), and *P-*values are derived from ANOVA and Tukey's multiple comparisons test.

Adult WT female mice received an orthotopic injection of murine breast cancer cells (Py230-luc) in the third mammary gland fat pad (Fig. 2A). Mice developed tumors, as indicated by the steady increase in the tumor volume (Fig. 2B–D) measured by the BLI and a digital caliper. Both methods of tumor measurements showed comparable tumor growth for the Py230 + Vehicle group (Fig. 2C–D). On the other hand, Py230 breast cancer-bearing mice receiving the adjuvant chemotherapy (ADR-CYP) starting from 10 days post-tumor induction showed significant reductions in tumor volume (Fig. 2B–D) indicating a therapeutic response of the tumor to the chemotherapy. Thus, this experimental design provides a testable breast cancer chemobrain model. About 8 weeks after the final chemotherapy injection, mice underwent the OLM testing, assessing the functional impact of cancer and chemotherapy on spatial recognition memory, which is dependent on intact hippocampal function. Cognitively intact mice will spontaneously spend more time exploring the novel placements of the object (Fig. 2E). The chemotherapy-treated cancer-bearing group (Py230+ADR-CYP) spent significantly less time exploring the novel placement when compared to the control group (P < 0.02), represented by a negative memory index (MI) (Fig. 2F).

#### Dynamin-1 expression reduced in the breast cancer-bearing mouse brain treated with adjuvant chemotherapy

**Fig. 3:**
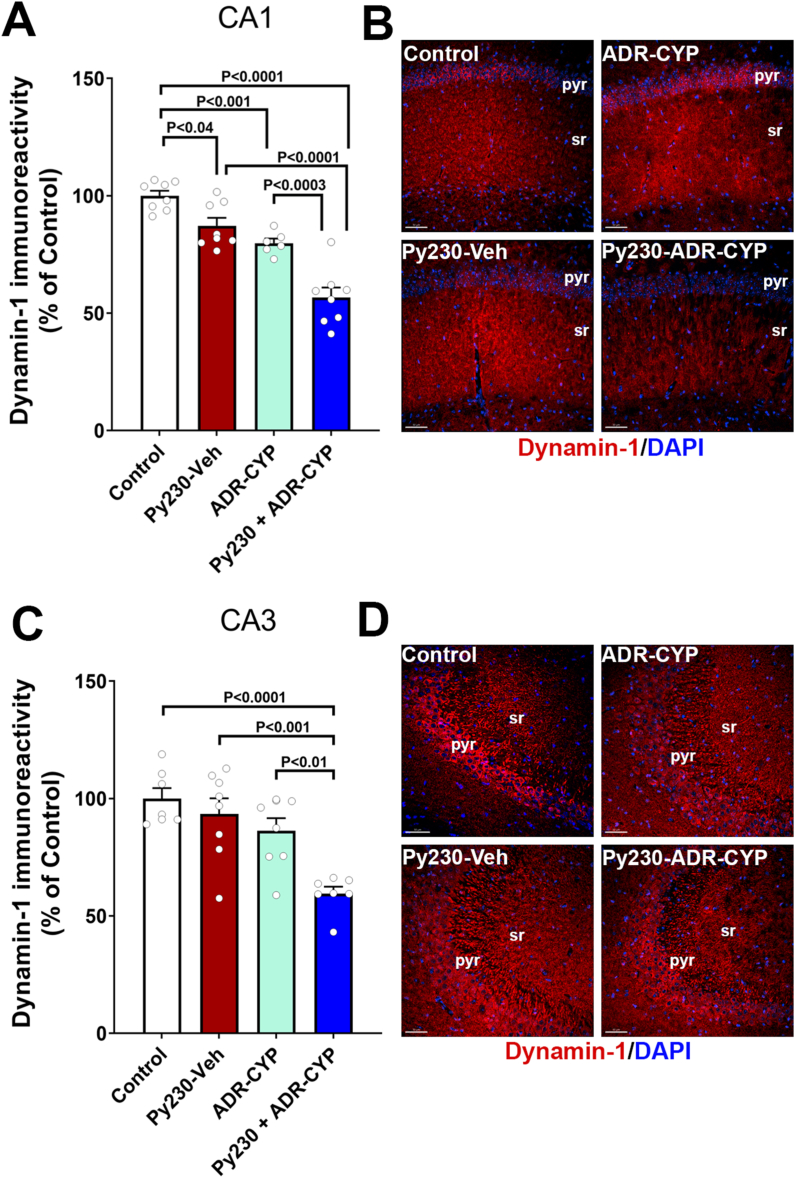
**Reduced hippocampal Dynamin-1 expression in a mouse breast cancer chemobrain model. (A**–**D)** Immunofluorescence staining for Dynamin-1 (red, DAPI nuclear counter stain, blue) showed reduced immunoreactivity in the noncancer (ADR-CYP) and cancer-bearing mice (Py230-Veh, Py230 ​+ ​ADR-CYP) receiving adjuvant chemotherapy at 8–9 weeks post-cancer induction compared to controls in the hippocampal CA1 and CA3 pyramidal layer (pyr) and stratum radiatum (sr). Breast cancer-bearing mice treated with ADR-CYP showed the most drastic decline in the Dynamin-1 immunoreactivity in the CA1 and CA3 subregions. Data are presented as mean ​± ​SEM (N ​= ​6–8 mice per group). ∗P values were derived from a two-way ANOVA and Tukey's multiple comparisons test. Scale bars, 50 ​μm ​**(B, D)**.

At 8–9 weeks post-tumor induction, mice were euthanized, and fixed brains were collected for the immunofluorescence staining and volumetric immunoreactivity quantification of DNM1 (Fig. 3A–D). Noncancer WT female mice treated with ADR-CYP were also included to compare DNM1 expression with the cancer-bearing mice. DNM1 expression was observed throughout the hippocampus in the control brains in the hippocampal CA1 and CA3 subregions. Particularly, DNM1 positive puncta were located within the pyramidal layer neuronal soma that emanated through the stratum radiatum (Fig. 3A and D). DNM1 positive immunoreactive puncta were quantified using a 3D algorithm-based surface rendering and volumetric quantification. In comparison with the control (no cancer) brains, breast cancer-bearing mice treated with vehicle (Py230-Veh), non-cancer mice treated with chemotherapy (ADR-CYP group), and breast cancer mice treated with ADR-CYP (Py230 ​+ ​ADR-CYP) had about 13 ​%, 20 ​%, and 43 ​% reduction respectively in the DNM1 immunoreactivity in the CA1 region (P ​< ​0.04, 0.001, and 0.0001, respectively, Fig. 3A). For the CA3 subregion (Fig. 3C), Py230 ​+ ​ADR-CYP group had the most significant decline in the DNM1 immunoreactivity compared to controls (P ​< ​0.0001, 40 ​% reduction), Py230-Veh (P ​< ​0.001, 33 ​% reduction), and noncancer ADR-CYP (P ​< ​0.01, 26 ​% reduction) mice brains. In total, breast cancer-bearing mice treated with adjuvant ADR-CYP showed the most drastic reductions in the DNM1 immunoreactivity in the hippocampal CA1 and CA3 subregions important for learning and memory functions.

## Discussion

In both humans and animals, we consistently observed that DNM1 downregulation could underlie CRCI pathogenesis. These findings are important and innovative in several ways. We are the first to quantify DNM1 levels within peripheral blood in human samples, and to add to the existing literature regarding the potential of EVs as biomarkers to research on cancer-related complications. Our human study was congruent with our previous study [12], with 46 ​% lower DNM1 expressions among cancer patients with CRCI compared to those without CRCI following exposure to neurotoxic chemotherapy. Ethnicity and fatigue were also reportedly associated with DNM1 levels in our multivariate analysis. Our breast cancer chemobrain animal model data has added new dimensions of evidence. We found the most drastic reductions in DNM1 immunoreactivity within hippocampal CA1 and CA3 subregions, and a consequential decline in spatial recognition memory among breast cancer-bearing mice treated with adjuvant chemotherapy. Taken together, this study provides important evidence that DNM1 is a biomarker associated with CRCI, and potentially a therapeutic target.

DNM1 plays a crucial role in ensuring effective neurotransmission by facilitating synaptic vesicle recycling via endocytosis, thus DNM1 depletion may reflect reduced neuronal activity dependent on synaptic vesicles [7,8]. DNM1 inhibition reduces hippocampal long-term potentiation and synaptic plasticity, suggesting its importance for the formation of associative memory in the hippocampus [7,28]. DNM1 also plays an important role in neurite development. Previous rodent studies have observed that decreased DNM1 expression cause defects in the biogenesis and endocytic recycling of synaptic vesicles, which impact the neuronal ability to regulate synaptic transmission [29]. Reduction of hippocampal DNM1 in aged mice was related to a decline in hippocampal-dependent memory [30]. There is literature suggesting that expression of DNM1 is associated with genetic polymorphism, with some rare neurological diseases found to be caused by DNM1 mutations [31]. Our previous pre-clinical studies have shown that in addition to reduced neurogenesis [32], chemotherapy had a detrimental impact on the hippocampal dendritic architecture and reductions in spine density. Particularly, chemotherapy significantly reduced immature spines in the hippocampal dentate gyrus and CA1 regions that plays vital roles in learning and memory function [33]. Taken together, DNM1 could be mediating the structural and functional alterations in the neuronal and synaptic landscape in the brain and contribute to CRCI pathogenesis.

Cancer patients require follow-up care for CRCI up to 10 years post-cancer diagnosis [34], yet there remains a lack of effective pharmacological management possibly due to poor understanding of the underlying CRCI mechanism [35]. The etiology of CRCI is likely multifactorial – growing literature suggests that reduction of brain derived neurotrophic factors is associated with CRCI, likely due to the reduction of synaptic plasticity as well as neurogenesis [36]. If cancer- and chemotherapy-induced reduction of DNM1 is truly linked with the long-term neurodegenerative consequences culminating into cognitive impairments, replacement of DNM1 may be another viable therapeutic strategy for ameliorating CRCI. In one study, investigators evaluated the effects of catalpol which was isolated from herbal medicine *Rehmannia glutinosa* and evaluated its impact on synaptic plasticity in aged rat models [37]. The investigators observed that catalpol markedly improved the cognitive function of aged male Sprague-Dawley rats and simultaneously increased the expression of synaptic proteins including DNM1, PSD-95, and synaptophysin in the cerebral cortex and hippocampus, respectively [37]. However, a series of systematic preclinical studies are still required to evaluate the therapeutic role of catalpol in mitigating CRCI. These studies should include breast cancer chemobrain models to establish the underlying mechanistic pathways. In addition, given that there are reports suggesting DNM1 may enhance cancer cell growth and tumor invasion, further studies are required [38].

There are key considerations when interpreting our findings. First, we have not associated objective cognition with DNM1 levels in humans, which is more widely recognized as a key cognitive endpoint relative to subjective cognition [2]. There is, however, growing literature to demonstrate the usefulness of subjective cognitive measures in epidemiological research and clinical settings [39]. Subjective cognition is arguably a more accurate reflection of how cognitive changes can affect daily functioning compared to objective cognition which is often criticized for the lack of ecological validity [39,40]. While subjective and objective cognitive function may not match for individual patients [[41], [42], [43]], biomarkers of CRCI, such as pro-inflammatory IL-6 and TNFα, as well as neurogenesis factor BDNF have found consistent directional correlations in studies utilizing both types of cognitive measures [16,36,44]. The biological underpinnings of both dimensions of cognition might be more similar than expected.

Our current cohort of cancer patients is significantly different in demographic (age and sex) and clinical (cancer diagnoses and anticancer treatment received) characteristics compared to our exploratory cohort of breast cancer patients [12]. Due to limited AYA cancer research and the rarity of this population, the cohort was initially established to assess the prevalence and incidence of CRCI in newly diagnosed AYA cancer patients. Eligibility criteria were strategically designed for age-based recruitment, rather than focusing on cancer or treatment types [15]. With the small sample size in the current analysis, we were unable to incorporate the different cancer-related covariates into the model. Nevertheless, this paper establishes the potential role of DNM1 in CRCI pathogenesis and we recommend that future studies investigate the impact of different cancer types and treatment regimens on DNM1 levels and CRCI in a more appropriate homogeneous cohort of cancer patients. Nonetheless, there are multiple merits with our human study; we have incorporated multiple assessment time points and a non-cancer control following recommendations provided by the International Cognition and Cancer Task Force to facilitate modeling of time-varying cognitive outcomes and isolation of cancer-specific mechanisms [14]. The purposeful recruitment of AYA cancer participants helped with minimizing the confounding effects from age-related neurodegenerative diseases. While some human data were collected during COVID-19 pandemic wherein social isolation and higher stress levels may have impacted DNM1 levels among non-cancer controls, our multivariable model has accounted for numerous factors including psychological distress and fatigue.

Finally, current literature is lacking with regards to the relationship between brain/hippocampal, plasma, and plasma EV DNM1 levels. In comparison to analyzing DNM1 directly in plasma, measuring it within EVs provides insights into the role of cell-to-cell communication, cellular processes and signaling pathways in the pathogenesis of CRCI [45]. In addition, as proteins encapsulated in EVs are stable and have a long half-life, EVs are a more preferred medium for quantifying proteomes in long-term stored biosamples [46,47]. Nevertheless, these elements will be incorporated in our future human and animal studies to further validate and understand the role of DNM1 in CRCI.

In conclusion, we found that downregulation of DNM1 is linked with the onset of CRCI and is consistent in both cancer patients and tumor-bearing mice receiving chemotherapy, strengthening our speculation on DNM1 role as a mediator for CRCI. Upcoming research work should include additional transdisciplinary studies to investigate key mechanisms and generate a clear pathway DNM1 modulation of cognitive function in CRCI trajectories. Further investigation on how this relationship may differ between subjective and objective cognitive impairment, and across different cancer and treatment types will also help to better contextualize the role of DNM1 in CRCI pathogenesis. DNM1 might be a biomarker and therapeutic target for CRCI.

## Data Availability

The datasets generated during and/or analyzed during the current study are available from the corresponding author on reasonable request.

## Author Contribution

Conceptualized and designed study: MMA, AC. Acquired and analyzed data: DQN, CH, TN, SKG, and YQK. Interpreted data, drafted, revised, and finalized manuscript: DQN, CH, TN, SKG, YQK, MMA, and AC. All authors have read and approved the manuscript for publication.

## Declaration of Competing Interest

We do not have any known competing financial interests or personal relationships that could have appeared to influence the work reported in this paper.
