# Supplementary material for: Dynamin-1 is a potential mediator in cancer-related cognitive impairment
Source: Neurotherapeutics. 2024 Nov 7;22(1):e00480. doi: 10.1016/j.neurot.2024.e00480 (PMC11742811; doi:10.1016/j.neurot.2024.e00480)
Supplement: Multimedia component 1 [file mmc1.docx]

**Supplement**

DQ Ng, C Hudson, T Nguyen, SK Gupta, YQ Koh, MM Acharya, A Chan. Dynamin-1 is a potential mediator in Cancer-Related Cognitive Impairment.

**Fig. S1: Distribution of Dynamin-1 levels at baseline, stratified by cancer status.**

**Table S1: Generalized estimating equation (GEE) output for predicting log-transformed Dynamin-1 levels**

**Fig. S1: Distribution of Dynamin-1 levels at baseline, stratified by cancer status.**

The figure illustrates the right-skewed distribution of Dynamin-1 levels at baseline for both non-cancer and cancer participants.

**Table S1: Generalized estimating equation output for predicting log-transformed Dynamin-1 levels**

|  | ***β*** | **Relative change *(exp(β))*** | **95% Confidence Intervals *(exp(β))*** | **p** |
| --- | --- | --- | --- | --- |
| Perceived cognitive decline (PCD)^a, b^ | -0.16 | 0.85 | 0.48 to 1.52 | 0.592 |
| PCD × Cancer^b^ | -0.46 | 0.63 | 0.29 to 1.35 | 0.233 |
| Baseline DNM1 | 0.01 | 1.01 | 1.01 to 1.01 | <0.001*** |
| Cancer | 0.71 | 2.04 | 1.51 to 2.77 | <0.001*** |
| Age (in years) | 0.03 | 1.03 | 1.00 to 1.06 | 0.065 |
| Years of education | 0.003 | 1.00 | 0.96 to 1.05 | 0.904 |
| Female (ref: Male) | 0.23 | 1.26 | 0.90 to 1.76 | 0.184 |
| Ethnicity |  |  |  |  |
| Chinese | ref | ref | ref | ref |
| Malay | -0.81 | 0.45 | 0.21 to 0.97 | 0.041* |
| Indian | 0.10 | 1.11 | 0.84 to 1.47 | 0.463 |
| Others | 0.53 | 1.70 | 1.12 to 2.57 | 0.013* |
| Married | 0.24 | 1.27 | 0.87 to 1.85 | 0.223 |
| Change in RSCL-PD from baseline (distress) | 0.04 | 1.04 | 0.98 to 1.10 | 0.161 |
| Change in MFSI-SF from baseline (fatigue) | -0.02 | 0.98 | 0.97 to 0.99 | 0.001** |
| Abbreviations: *β*, coefficient; *exp(β)*, DNM1, Dynamin-1; exponential of the coefficient; MFSI-SF, Multidimensional Fatigue Symptom Inventory-Short Form; PCD, perceived cognitive decline; ref, reference; RSCL-PD, Rotterdam Symptom Checklist – Psychological Distress.  ^a^ The *exp(β)* represents the relative difference in DNM1 levels between PCD and non-PCD among non-cancer controls. The explanations are as follows:   - PCD, non-cancer: *log(E(DNM1))* = [other covariates] + ***β_PCD_*** + ε. - Non-PCD, non-cancer: *log(E(DNM1))* = [other covariates] + ε. - Thus, ***exp(β_PCD_)*** represents the relative differences in DNM1 levels between PCD and non-PCD participants among non-cancer controls in this study. The statistics are presented in Tabel 2 – Model A.   ^b^ The exponential of the linear combination of *β* for PCD and PCD × Cancer represents the relative difference in DNM1 levels between PCD and non-PCD among cancer participants. The explanations are as follows:   - PCD, cancer: *log(E(DNM1))* = [other covariates] + β_cancer_ + ***β_PCD × Cancer_ + β_PCD_*** + ε. - Non-PCD, cancer: *log(E(DNM1))* = [other covariates] + β_cancer_ + ε. - Thus, ***exp(β_PCD × Cancer_ + β_PCD_)*** represents the relative differences in DNM1 levels between PCD and non-PCD participants among cancer patients in this study. The statistics are presented in Table 2 – Model A.   * p < 0.05; ** p < 0.01; *** p < 0.001. | | | | |
